# Bacteria isolated from the grape phyllosphere capable of degrading guaiacol, a main volatile phenol associated with smoke taint in wine

**DOI:** 10.1371/journal.pone.0331854

**Published:** 2025-10-01

**Authors:** Claudia Castro, Jacquelyn Badillo, Melissa Tumen-Velasquez, Adam M. Guss, Thomas S. Collins, Frank Harmon, Devin Coleman-Derr

**Affiliations:** 1 Plant Gene Expression Center, United States of America Department of Agriculture - Agricultural Research Service, Albany, California, United States of America; 2 Department of Molecular and Cell Biology, University of California, Merced, Merced, California, United States of America; 3 Biosciences Division, Oak Ridge National Laboratory, Oak Ridge, Tennessee, United States of America; 4 Viticulture and Enology Program, Washington State University, Richland, Washington, United States of America; Institute for Sustainable Plant Protection, C.N.R., ITALY

## Abstract

Recent wildfires near vineyards in the Pacific United States have caused devastating financial losses due to smoke taint in wine. When wine grapes (*Vitis vinifera*) are exposed to wildfire smoke, their berries absorb volatile phenols derived from the lignin of burning plant material. Volatile phenols are released during the winemaking process giving the finished wine an unpleasant, smokey, and ashy taste known as smoke taint. Bacteria are capable of undergoing a wide variety of metabolic processes and therefore present great potential for bioremediation applications in many industries. In this study, we identify two strains of the same species that colonize the grape phyllosphere and are able to degrade guaiacol, a main volatile phenol responsible for smoke taint in wine. We identify the suite of genes that enable guaiacol degradation in *Gordonia alkanivorans* via RNAseq of cells growing on guaiacol as a sole carbon source. Additionally, we knockout *guaA,* a cytochrome P450 gene involved in the conversion of guaiacol to catechol; Δ*guaA* cells cannot catabolize guaiacol *in vitro,* providing evidence that GuaA is necessary for this process. Furthermore, we analyze the microbiome of berries and leaves exposed to smoke in the vineyard to investigate the impact of smoke on the grape microbial community. We found smoke has a significant but small effect on the microbial community, leading to an enrichment of several genera belonging to the Bacilli class. Collectively, this research shows that studying microbes and their enzymes has the potential to identify novel tools for alleviating smoke taint.

## Introduction

Vegetation fires are an ancient and essential component of the Earth system, and recent changes in their frequency have resulted in significant agricultural losses [1]. One consequence of these changes is that wildfires have become more frequent and unpredictable in the Pacific United States. Besides the destruction of infrastructure and environmental damage, wildfires have an unexpected and indirect negative impact on the production of wine grapes and their vinification. When wine grape berries are exposed to high levels of smoke in the vineyard, they absorb volatile phenols present in smoke causing an unwanted change in the chemistry profile of the fruit [2–7]. The absorbed volatile phenols (i.e., guaiacol, 4-methylguaiacol, syringol, etc.) become glycosylated by the grape leaves and berries and result in a final wine product with unpleasant flavors described as ashy, smokey in taste [2–8]. This impact on wine, known as smoke taint, was first described in the wine-growing regions of Australia and has recently become a major issue in vineyards of the Western United States [2,9,10]. Smoke taint in wine has caused significant economic losses for the wine industry in recent years. For example, California and Oregon lost $3.7 billion due to smoke taint in 2020 [11]. As a result, the wine industry is in dire need of new technology to help alleviate this challenging biochemical problem.

Crop plants, including grapevines, have microbial cells living in direct contact with them that provide many services to their host such as protection against pathogens, drought tolerance, and nutrient uptake. These communities are dynamic and respond to environmental changes like abiotic or biotic events. Upon these environmental changes, the microbiome can shift in terms of cell number or species presence. Following wildfire, high levels of smoke can create a harsh environment for the grape microbiome, including low moisture, low oxygen, and aerosolized burnt particles that will alter the metabolic landscape at the grape or grape leaf surface. We hypothesize that these changes will potentially impact the microorganisms living on the surface of aerial tissues of their host. Although the grape microbiome has been described by several research groups, not many studies have focused on the exploration of specific metabolic functions of individual members of the grape microbiome [12–15].

Bacteria possess a wide range of metabolic capabilities making them great resources for the development of new tools for the food, pharmaceutical, agricultural, and biofuel industries. In the case of smoke taint, microbial enzymes that catabolize aromatic compounds for the bioremediation of affected berries could be of great value to the wine industry and are currently of particular interest. The volatile phenol guaiacol is one of the dominant volatiles found in wildfire smoke, and is also commonly reported among the most abundant aromatic compounds in tainted wine [2,3,6]. Although guaiacol is typically toxic to bacteria, a few bacterial species have been reported to possess the ability to degrade this compound [16–20]. However, studies that have focused on the microbial enzymes involved in guaiacol catabolism are rare; in three recent, independent studies on this topic, several Gram positive bacteria typically found in soil (*Rhodococcus rhodochrous, Rhodococcus opacus,* and *Amycolatopsis* sp.) were shown to utilize a cytochrome P450 monooxygenase and an associated redox partner to convert guaiacol to catechol by demethylation. Catechol is further broken down via the β-ketoadipate pathway and converted in end-products succinyl-CoA and acetyl-CoA to be used in the TCA cycle or in fatty acid biosynthesis [21–23]. Whether these or related enzymes with the ability to degrade guaiacol are present in the grape microbiome remains unknown.

In this study, we identify bacteria within the grape microbiome capable of degrading guaiacol and identify changes in the grape microbiome caused by simulated wildfire smoke with the goal of providing genetic tools to help combat smoke taint in wine. We describe the discovery of two grape associated bacterial strains of the same species, *Gordonia alkanivorans*, that can assimilate guaiacol *in vitro*. Furthermore, we analyze the transcriptomes of these strains growing on guaiacol as a sole carbon source and identify the genes involved in the degradation of this compound. Through genetic knockout in one of these strains, we show that the cytochrome P450 GuaA is necessary for guaiacol degradation. Finally, we demonstrate that smoke exposure has a significant but transitory impact on the bacterial communities of grape leaves and berries in the vineyard, demonstrating the potential that smoke exposure has for altering the landscape of protective plant microbe associations present there.

## Materials and methods

### Leaf isolates assays for bacteria capable of utilizing guaiacol as a sole carbon source

Two individual leaves from *Vitis vinifera* ‘Chardonnay’ and ‘Cabernet Sauvignon’ plants grown in a greenhouse (Albany, CA, USA) were collected and placed in sterile 50 mL conical tubes containing 3 mL of M9 salts medium without a carbon supplement. Samples were vortexed for one minute at medium speed to remove leaf epiphytes. 500 mL of the resultant leaf wash was added to 4 mL liquid M9 salts medium (BD Difco M9 Minimal Salts 5X, Cat. No. 248510) supplemented with either glucose (22 mM), guaiacol (4 mM), or no carbon source. Liquid cultures were then incubated at 30°C, 250 rpm for 7 days. 200 μL of growth were subcultured into fresh 4 mL M9 medium of the corresponding type and these new cultures were incubated 30°C, 250 rpm for 5 days. Serial dilutions were performed and plated on tryptic soy agar (TSA) medium to isolate bacteria growing in M9 + guaiacol as mixed cultures. Individual colonies were checked for growth in M9 + guaiacol again as axenic cultures.

### Species identification

Two orange-colored isolates, Vvg01 and Vvg02, were grown on tryptic soy broth 30°C, 250 rpm for 4 days, after which cells were harvested and used for high molecular weight DNA extraction with the Monarch® HMW DNA Extraction Kit for Tissue (#T3060L, New England Biolabs, Ipswich, MA, USA). Primers 27F and 1492R were used to amplify the full length 16 rRNA region with Platinum Hot Start PCR 2X Master Mix (#13000014, Invitrogen, Waltham, MA, USA). The resulting PCR product was submitted for Sanger sequencing and the assembled contig was screened against the NCBI nucleotide database using BLAST for isolate identification.

### Isolate genome sequencing

High molecular weight DNA from isolates Vvg01 and Vvg02 was submitted to Novogene Corporation Inc. (Sacramento, CA, USA) for whole genome sequencing on the PacBio Sequel II system (Menlo Park, CA, USA). Sequencing libraries were prepared according to the protocol provided by Pacific Biosciences of California (Template Preparation and Sequencing guide 2010–2014). Reads were filtered and processed with SMRT Link v12.0n (pacb.com/support/software-downloads) with ‘minLength 0’ and ‘minReadScore 0.8’ as parameters. Sequencing for Vvg02 resulted in a total of 30,275 reads with a mean length of 61,724 and a *N*_*50*_ value of 95,254. Sequencing for Vvg01 resulted in a total of 29,906 reads with a mean length of 61,450 and a *N*_*50*_ value of 94,530. *De novo* long-read genome assembly was performed on subreads using Falcon v1.8.1 [24]. Bacterial taxonomic classification was performed using GTDB-Tk v2.2.6 [25]. Functional annotation was performed using GO, KEGG, KOG, NR and Pfam databases and results were filtered by selecting the one with the highest score. Details on genome features are reported in Table 1.

**Table 1:** Genome features for guaiacol-degrading bacterial isolates.

	Vvg01		Vvg02	
**Genome features**	**Chromosome**	**Plasmid**	**Chromosome**	**Plasmid**
Source	*Vitis vinifera* (CA, USA)		*Vitis vinifera* (CA, USA)	
No. of reads	178,430		201,784	
%GC	67	66	67	66
Assembly size (bp)	5,135,626	128,280	5,078,688	138,437
No. of contigs	1	1	1	1
No. of coding genes	4,734	132	4,648	141
GTDB assign taxon	*Gordonia alkanivorans*		*Gordonia alkanivorans*	
GTDB accession	GCF_000225505.1		GCF_000225505.1	
GTDB FastANI score	98.35%		98.14%	

Our analysis predicted a total of 4,866 and 4,789 genes were predicted for Vvg01 and Vvg02, respectively. These strains were assigned as *Gordonia alkanivorans* species based on average nucleotide identity (ANI) scores of 98.14% and 98.35% for Vvg01 and Vvg02.

### *In vitro* growth dynamics

Vvg01 and Vvg02 strains were grown on TSA medium at 30°C for 5 days. Two colonies of similar size were inoculated onto 4.5 mL M9 + guaiacol (4mM) liquid medium (n = 12). M9 medium without a carbon source served as a negative control and M9 + guaiacol without any bacteria was used as a blank. Cultures were incubated at 30°C, 250 rpm and samples were collected every 24 hrs. 100 μL was used for growth measurements (OD_600 nm_) and 500 μL for guaiacol quantification. The samples for guaiacol quantification were centrifuged 6,000xg for 5 min. The supernatant was filter sterilized with a syringe filter (0.22 μm) and stored at −20°C. The Phenolic Compounds Assay Kit (ab273293, Abcam, Cambridge, UK) was used to quantify guaiacol presence in bacterial cultures. This assay can detect concentrations of phenolic compounds as low as 0.02 mM.

### Transcriptional analysis

*Gordonia alkanivorans* strains Vvg01 and Vvg02 were grown on M9 + guaiacol (4 mM) or M9 + mannitol (22 mM) liquid medium until the cultures reached the exponential growth phase, 48 hours post-incubation (n = 5). Bacterial cultures were centrifuged in a 15 mL conical tube at 4,000xg, 4°C for 5 min. Supernatant was quickly removed and cell pellets were immediately frozen in liquid nitrogen and stored at −80°C. Total RNA was purified using the Zymo Quick RNA Fungal/Bacterial Miniprep kit (R2014, Zymo, Irvine, CA, USA). RNA samples were submitted to Novogene Corporation Inc. (Sacramento, CA, USA) for sequencing. Ribosomal RNA was removed from total RNA using the Illumina Ribo-Zero PLus rRNA Depletion Kit (20040526, Illumina, San Diego, CA, USA) and cDNA libraries were made using NEBNext Ultra II RNA Library Prep Kit for Illumina (#E7775, New England Biolabs, Ipswich, MA, USA). cDNA libraries were sequenced using an Illumina Novaseq sequencer (Illumina, San Diego, CA, USA) as 150 bp paired-end reads. Raw reads were pre-processed with HTStream v1.3.3 (https://github.com/s4hts/HTStream). This processing removed extraneous sequence features and contaminants, such as Illumina adapters, PhiX contamination, reads fewer than 50 basepairs in length, and basepairs at the ends of reads whose average quality score was less than 20. Reads were mapped onto the respective predicted protein-coding sequences of *Gordonia alkanivorans* Vvg01 and Vvg02 using STAR v2.7.10b [26]. Counts of uniquely mapped reads were generated using STAR v2.7.10b. Read mapping results are reported in S1 File. The Bioconductor R package DESeq2 v1.36.0 [27] was used to normalize read counts and to determine differentially expressed genes (DEGs, **adj. P-value* *< 0.05 and log_2_FC ≥ 1.5).

### Time course RNAseq experiment

Vvg01 strain was grown on TSA medium at 30°C for 5 days. A single colony from this plate was inoculated into 4 mL M9 + mannitol (22 mM) liquid medium and incubated at 30°C, 250 rpm for 48 hrs. Cells were centrifuged at 4000xg for 5 min. at room temperature. The supernatant was decanted and the cell pellet was washed with 500 μL of M9 salts. Wash step was repeated twice to remove all remaining M9 + mannitol medium from cells. After the wash, the cell pellet was resuspended in 100 μL M9 salts and inoculated into fresh 4 mL of either M9 + mannitol (22 mM) or M9 + guaiacol (4 mM) liquid medium. Bacterial cultures were incubated again at 30°C, 250 rpm until harvest for RNA extraction at time points 20, 60, and 300 minutes after incubation (n = 4 per time point). Cells were harvested, flash frozen, and had their RNA extracted as mentioned above. RNA samples were submitted to the UC Davis Genome Center DNA Technologies & Expression Analysis Core Laboratory for library preparation and sequencing. Strand-specific and dual-barcode indexed RNA-seq libraries were generated from 300 ng total RNA each using the Kapa RNA-seq Hyper kit (Kapa Biosystems-Roche, Basel, Switzerland) and the QIAseq FastSelect–5S/16S/23S ribodepletion reagent (Qiagen, Hilden Germany) in combination following the instructions of the manufacturers. The fragment size distribution of the libraries was verified via micro-capillary gel electrophoresis on a LabChip GX system (PerkinElmer, Waltham, MA). The libraries were quantified by fluorometry on a Qubit instrument (Life Technologies, Carlsbad, CA) and pooled in equimolar ratios. The pool was quantified by qPCR with a Kapa Library Quant kit (Kapa Biosystems) and sequenced on an Illumina NovaSeq X 25B (Illumina, San Diego, CA) with paired-end 150 bp reads. Read mapping results are reported in S2 File. Raw reads processing and differential expression analysis were done as mentioned above.

### *guaA* gene knockout

To prepare electrocompetent cells, *G. alkanivorans* was grown on TSA for five days at 30°C. A single colony was inoculated in 5 mL LB and incubated for two days at 30°C, 250 rpm. 1 mL of this culture was added to 100 mL modified LB (1% glycine w/v, 1.5% sucrose w/v, 2% Tween 80, 3 μg/mL isonicotinic acid hydrazide added after autoclaving) in a culture flask. Cultures were incubated until cells reached exponential phase, OD_600 nm_ of ~0.5. This culture was divided into two 50 mL conical tubes and placed on ice for 30 min. Cells were centrifuged for 5 min at 4°C, 4,000 rpm. Supernatant was discarded and cell pellets were gently washed with 15 mL cold 10% glycerol in water. Wash step was repeated twice. Finally, both cell pellets were gently resuspended in 1 mL cold 10% glycerol and stored in −80°C as 100 μL aliquots until use. All bacterial strains, plasmids and primers are listed in S3 File. The remaining methods for this section can be found in S9 File under supporting information.

### Smoke exposure and sample preparation

Berry and leaf samples were collected from a *Vitis vinifera* ‘Merlot’ vineyard at the Washington State University Roza Farm in Prosser, Washington, USA in the year 2022 and the smoking was conducted August 13 and 14, 2022. The vines were planted in 2010 with spacing at six intervals along the rows which have 9 ft. spacing. Three portable hoop-houses containing 30 vines each were exposed to smoke for 36 hrs. and three hoop-houses with 30 vines each left untreated served as the control. The portable hoop-houses were 30 by 4.5 meters in dimension. They were covered with 80/20 shade cloth which contains the smoke but keeps the interior of the houses at ambient temperature. Smoke was generated using hardwood pellets (Traeger “Winemaker’s Reserve”) in an Oklahoma Joe side firebox smoker. A full tube of pellets generated smoke for 5 to 6 hrs. Smoke was delivered to the fruiting zone in each vineyard row using a 4 in ABS plastic drain line with 2.5 mm diameter holes drilled every 25 cm along the length of the line. An inline duct fan was used to move the smoke from the smoker into the distribution line. Eight evenly spaced vines were selected per plot, and berries and leaves were collected at three time points: before smoke, 36 hours post-smoke, 1 week post-smoke, 1 month post-smoke, and 2 months post-smoke treatment for a total of 240 berry and 240 leaf samples. All samples were stored at -80°C or placed on dry ice during transportation to the lab. Berries were lyophilized for 48 hrs. prior to maceration using a VirTis Freezemobile 25XL lyophilizer (SP Industries, Warminster, PA, USA). 100 to 150 mg frozen berry or leaf tissue was ground in a sterile 2 mL screw cap vial (Cat. No. 1420-9710, USA Scientific) with a steel bead (5/32” in., BC Precision) using a BioSpec Mini-Beadbeater-96 (Cat. No. 1001, Biospec, Bartlesville, OK, USA) at 2,000 rpm for 40 s with a frozen aluminum vial rack (Cat. No. 702ALU, Biospec, Bartlesville, OK, USA) to keep samples frozen. The remaining methods for this section can be found in S9 File under supporting information.

### 16S library preparation and bacterial community analysis

Dual-indexed primers for Illumina sequencing (341F: 5’- CCTACGGGNBGCASCAG-3’ and 785R: 5’-GACTACNVGGGTATCTAATCC-3’) were used to amplify the V3-V4 region of the 16S rRNA gene with 10 ng of template DNA and Invitrogen Platinum Hot Start PCR Master Mix (2X, Catalog No. 13000014, Waltham, Massachusetts, USA) along with 0.4 μg bovine serum albumin and host plastid and mitochondrial clamps (0.75 μM final each, PNA Bio., Thousand Oaks, CA, USA). PCR reactions were performed in triplicate with the following settings: 98 °C for 180 s, 30 cycles of 98 °C for 45 s, 78 °C for 10 s, 55 °C for 60 s, 72 °C for 90 s, and a final amplification of 72 °C for 10 min. PCR product was quantified using a Qubit 3.0 fluorometer (Invitrogen, Waltham, MA, USA) and Qubit 1X dsDNA High Sensitivity Assay Kit (Cat. No. Q33266, Invitrogen). 100 ng of PCR product for each sample was pooled. 1 μg of the pool was cleaned using Agencourt AMPureXP beads (Beckman-Coulter, West Sacramento, CA, U.S.A.) with the following protocol modifications: 1.5X volume beads were added, sample was washed twice with 200 μL plus the sample volume 70% ethanol and resuspended in 30 μL sterile nuclease-free water. The final pooled sample was quantified with a Qubit 3.0 fluorometer (Invitrogen, Waltham, MA, USA) and Qubit 1X dsDNA High Sensitivity Assay Kit (Cat. No. Q33266, Invitrogen). The final concentration was adjusted to 10 nM for sequencing at the University of California, Berkeley Vincent J. Coates Genomics Sequencing Laboratory using an Illumina MiSeq platform with kit v3 (Illumina, San Diego, CA, USA) to generate 300PE reads.

Raw reads were demultiplexed using QIIME2 [28] and processed using DADA2 [29] to create amplicon sequence variants (ASVs). Prior to removal, the total number of reads were 88.24% and 88.90% chloroplast and mitochondrial origin for berry and leaf samples. Samples with fewer than 1,000 clean reads were discarded. This resulted in 19,444 and 19,124 ASVs for grape berry (204 samples) and leaf (162 samples) tissues, respectively. Taxonomies were assigned using the SILVA SSU database 138 (released on December 16, 2019) of the 16S rRNA V3-V4 gene regions as a classifier trained in QIIME2 [28]. ASV tables were imported into R (v4.2.3). All reads taxonomically assigned as ‘chloroplast’ and ‘mitochondria’ were removed and samples resulting in fewer than 1,000 read counts were removed. R package phyloseq v1.40.0 [30] was used to determine alpha and beta diversity. For the alpha diversity, the number of reads per sample was rarefied to even depth and the diversity measure ‘Shannon’ was used. Tukey’s HSD was used to detect significance between comparisons. Beta diversity was analyzed via principal coordinates analysis (PCoA) using Bray-Curtis dissimilarity distance and a constrained analysis of principal coordinates on Bray-Curtis distances was performed with the variable ‘Treatment’ to constrain the ordination. Differences between sample groups was determined by a PERMANOVA. Differentially abundant taxa at the genus level were determined using the R package DESeq2 v1.36.0 [27]. Prior to analysis, a pseudocount of 1 was added to the ASV counts table.

## Results

### Grape bacterial isolates grow on guaiacol as sole carbon source

To identify bacterial species that could potentially degrade guaiacol, a major phenolic compound found in smoke tainted wine, we tested leaf epiphytes found in *Vitis vinifera* ‘Cabernet Sauvignon’ and ‘Chardonnay’ for their ability to grow in M9, a minimal and defined medium, supplemented with guaiacol as the sole carbon source. Leaf epiphytes were inoculated into M9 + guaiacol (4 mM) liquid medium as mixed cultures. After serial dilution and seven days of incubation on TSA plates, eight bacterial colonies with distinct morphology appeared and each one was individually tested for growth in guaiacol. Two candidates, both bright orange-colored colonies, were found to be able to grow in liquid M9 + guaiacol as axenic cultures (S1 Fig).

Despite differences in mucoidy and autoaggregation, Sanger sequencing of the 16S ribosomal RNA genes determined that both isolates were close relatives of *Gordonia alkanivorans*, a Gram-positive bacterium whose genus is known for their diverse metabolic capabilities such as the degradation of xenobiotic compounds [31–33]. Because taxonomic classification via 16S rRNA sequencing offers limited resolution at the specific epithet level and we were interested in exploring these strains’ genetic and functional capacity, we sequenced the genomes for these isolates via the PacBio platform. Assembly and annotation of the long read sequencing results determined that the genomes were non-identical but of similar size, with each containing a circular chromosome and single plasmid (Table 1). A total of 4,648 and 4,734 genes were predicted for Vvg01 and Vvg02, respectively. The assembled genomes were analyzed using GTDB-Tk [25,34] to assign taxonomy to prokaryotic species. This analysis confirmed that that the closest species to both isolates, assigned Vvg01 and Vvg02, was *G. alkanivorans* (GTDB accession GCF_000225505.1) with an average nucleotide identity (ANI) score of 98.14% and 98.35% (Table 1). As the ANI score for bacterial speciation threshold is 95%, our findings further confirmed our isolates to be strains belonging to *G. alkanivorans.*

### *Gordonia alkanivorans* isolates degrade guaiacol *in vitro*

**Fig 1:**
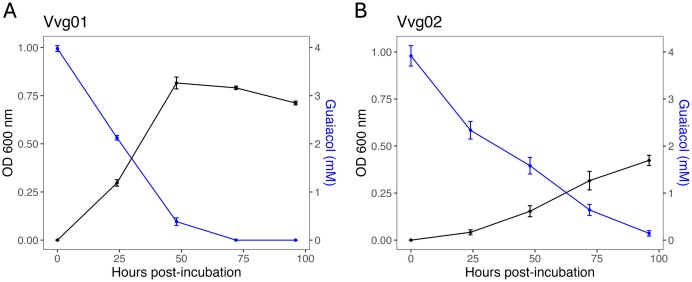
*Gordonia alkanivorans* strains isolated from *Vitis vinifera* ‘Cabernet Sauvignon’ and ‘Chardonnay’ leaves assimilate guaiacol *in vitro.* Bacterial growth (black line) and guaiacol quantification (blue line) in liquid M9 medium supplemented with guaiacol (4 mM) as the only carbon source for strains **(A)** Vvg01 and **(B)** Vvg02 (**n* *= 12)*.* Error bars represent standard error.

To provide further evidence that our *G. alkanivorans* isolates are capable of utilizing guaiacol as a carbon source for growth, we measured their growth on M9 + guaiacol (4 mM) along with quantification of guaiacol over time. Both isolates displayed the typical logarithmic bacterial growth *in vitro* and we observed that guaiacol diminished in the growth medium over time (Fig 1). After 72 hours of incubation, the guaiacol present in the Vvg01 cultures was below the detection limit (0.02 mM) of our assay while the Vvg02 cultures took approximately 24 hours longer to get to guaiacol levels under the detection limit. In addition, we tested Vvg01 and Vvg02 for growth on similar volatile phenols to guaiacol: phenol, 4-methylguaiacol, eugenol, 4-ethylguaiacol, syringol, and 4-ethylphenol. No growth was observed for either strain after seven days of incubation (S2 Fig). Collectively, these findings demonstrate both isolates are capable of using guaiacol as a carbon source for growth but not related phenolic compounds.

### A cytochrome P450 gene is highly upregulated upon growth on guaiacol

**Fig 2:**
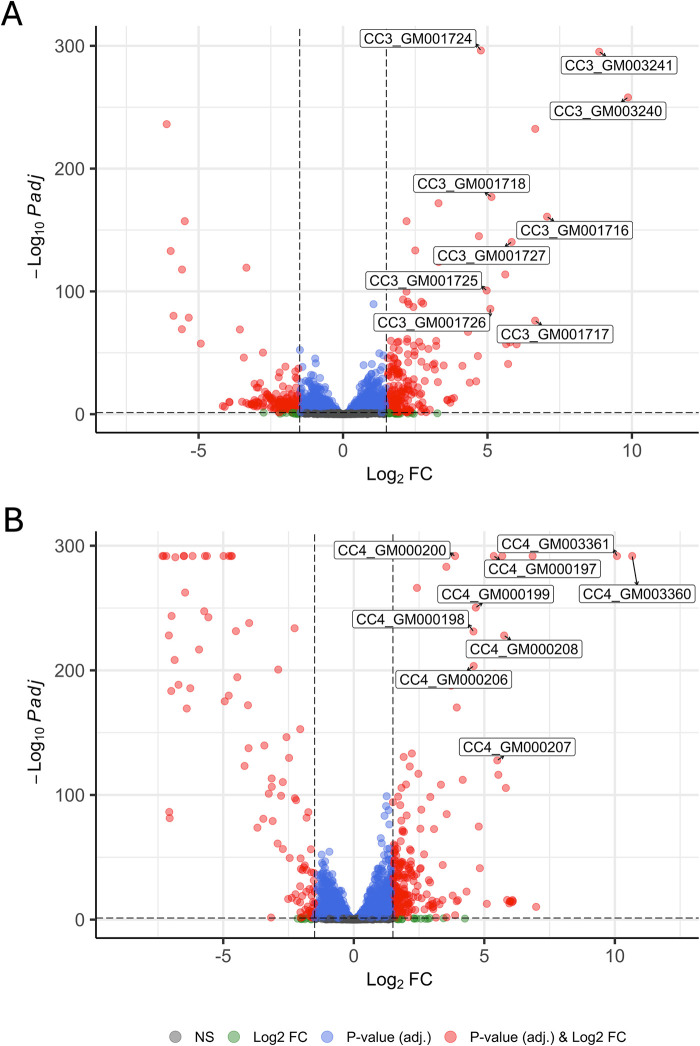
Genes predicted to participate in guaiacol degradation via the β-ketoadipate pathway were differentially expressed and highly up-regulated in both *Gordonia alkanivorans* strains during the exponential growth phase in response to guaiacol. These nine genes are labeled (boxed) for strain **(A)** Vvg01 and **(B)** Vvg02 and include a putative *O*-demethylase cytochrome P450 and reductase pair for the degradation of guaiacol named *guaA* and *guaB* (*CC3_GM003241* and *CC3_GM003240*; CC4*_GM003360* and *CC4_GM003361*). Vertical dashed lines indicate a threshold of log_2_FC ≥ 1.5 and the single horizontal dashed line indicates a threshold for adj. *P*-value < 0.05 (DESeq2, *n *= 5).

To identify the genes and pathways responsible for guaiacol assimilation in *G. alkanivorans,* we performed RNAseq of bacterial cells grown to the exponential phase in minimal media supplemented with either guaiacol or mannitol as the sole carbon source. When comparing treatments, expression analysis resulted in 363 and 305 differentially expressed genes (DEGs, DESeq2, log_2_FC ≥ 1.5 and *P.* adj. < 0.05) for strain Vvg01 and Vvg02, respectively (S5 and S6 Files). Among these, we identified a group of nine genes that were highly upregulated upon guaiacol growth and whose annotation indicated similarity to known proteins involved in guaiacol degradation (Fig 2 and Table 2). Based on our analysis of the annotations of most closely related homologs, we anticipated that these genes were responsible for the conversion of guaiacol to catechol, as well as the subsequent breakdown of this molecule into succinyl-CoA and acetyl-CoA via the β-ketoadipate pathway. Perhaps most significantly, the two most upregulated genes in this list (for both strains) were found to be genomically adjacent to one another, likely transcribed together in an ORF, and to share homology with genes coding for aromatic *O*-demethylase cytochrome P450 subunit and an aromatic *O*-demethylase reductase subunit (*CC3_GM003241* and *CC3_GM003240*; *CC4_GM003360* and *CC4_GM003361,* Table 2). Additionally, we found a putative transcriptional regulator gene belonging to the AraC family to be significantly upregulated in both Vvg01 and Vvg02 strains, *CC3_GM003242* and *CC4_GM003359,* hereafter referred to as *guaR*. This gene is also adjacent to and on the opposite DNA strand of the aromatic *O*-demethylase cytochrome P450 and reductase genes suggesting it may serve as the transcriptional regulator for *CC3_GM003241* and *CC3_GM003240*, and *CC4_GM003360* and *CC4_GM003361* (hereafter referred to as *guaA* and *guaB,* respectively). *GuaAB* are identical in sequence in both Vvg01 and Vvg02 and share similar genomic organization and protein sequence with the characterized guaiacol demethylation genes *gcoAB* in *Rhodococcus opacus* PD630 and *Amycolatopsis* sp.

**Table 2:** Genome annotations for the genes predicted to participate in guaiacol degradation via the β-ketoadipate pathway in *Gordonia alkanivorans* strains illustrated in Fig 2.

	Vvg01			Vvg02		
**Annotation**	**Log**_**2**_ **FC**	**Adj. P-value**	**Gene ID**	**Log**_**2**_ **FC**	**Adj. P-value**	**Gene ID**
Aromatic O-demethylase, reductase subunit	9.86	9.92E-259	CC3_GM003240	10.08	0.00E + 00	CC4_GM003361
Aromatic O-demethylase, cytochrome P450 subunit	8.87	6.32E-296	CC3_GM003241	10.66	0.00E + 00	CC4_GM003360
Chlorocatechol 1,2-dioxygenase	6.65	6.82E-77	CC3_GM001717	5.50	1.73E-128	CC4_GM000207
L-Ala-D/L-Glu epimerase	5.14	1.18E-177	CC3_GM001718	4.59	3.30E-204	CC4_GM000206
Muconolactone Delta-isomerase	7.06	1.42E-161	CC3_GM001716	5.76	1.10E-228	CC4_GM000208
3-oxoadipate enol-lactonase 2	5.10	1.68E-86	CC3_GM001726	4.58	6.07E-232	CC4_GM000198
Beta-ketothiolase BktB	5.84	5.66E-141	CC3_GM001727	5.36	0.00E + 00	CC4_GM000197
Putative succinyl-CoA:3-ketoacid coenzyme A transferase subunit A	4.97	1.92E-101	CC3_GM001725	4.67	4.01E-251	CC4_GM000199
Putative succinyl-CoA:3-ketoacid coenzyme A transferase subunit B	4.77	0.00E + 00	CC3_GM001724	3.88	0.00E + 00	CC4_GM000200

The annotations listed were determined with the software tool Prokka.

ATCC39116 (S7 File) [17–19,35]. Given these results, we hypothesize the *G. alkanivorans* genes *guaA* and *guaB* are transcriptionally regulated under *guaR* and are responsible for the conversion of guaiacol to catechol.

Among the differentially expressed genes for strain Vvg01, we detected one additional gene predicted to encode a cytochrome P450, *CC3_GM001521*. However, this was expressed at a much lower level compared to the highly upregulated *guaA* cytochrome P450, log_2_FC of 2.25 *versus* 8.87, respectively. This gene does not have a neighboring reductase gene partner nor a transcriptional regulator gene making it an unlikely candidate for a guaiacol *O*-demethylase. In regards to our DEG analysis for strain Vvg02, we did not detect any additional cytochrome P450 genes as differentially expressed making *guaA* the likely cytochrome P450 for guaiacol degradation. In addition, we found three genes related to transport that were upregulated in both strains (*CC3_GM001949*, *CC3_GM002933,* and *CC3_GM003855* in strain Vvg01 and *CC4_GM002767*, *CC4_GM004221*, and *CC4_GM004639* for strain Vvg02). Both strains Vvg01 and Vvg02 had two upregulated genes in common that were homologs to *Pseudomonas putida* BenE and BenK, two transporters responsible for benzoate uptake (Vvg01 *CC3_GM001949* and *CC3_GM003855,* Vvg02 *CC4_GM002767* and *CC4_GM004639*) [36,37].

**Fig 3:**
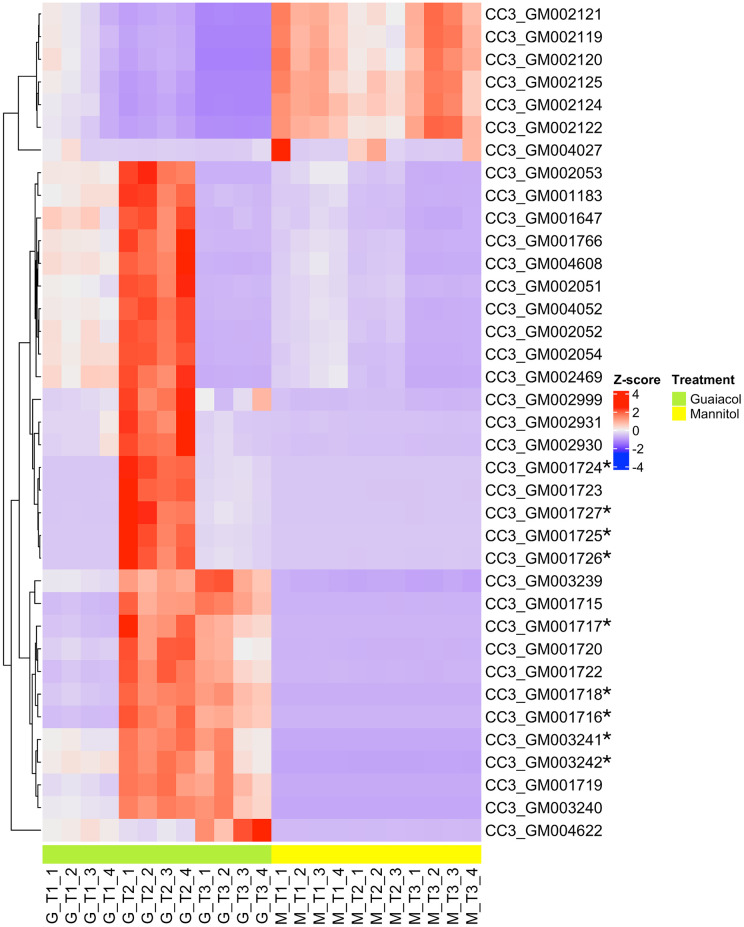
Genes predicted to participate in guaiacol degradation are significantly upregulated during early growth in liquid M9 supplemented with guaiacol as the sole carbon source. *Gordonia alkanivorans* Vvg01 cells were grown on mannitol (yellow samples) then transferred to grow on guaiacol (green samples), with both substrates used as the sole carbon source. After this transfer, cells were collected at 20 min. (T1), 60 min. (T2), and 300 min. (T3) post-incubation for RNASeq analysis. The nine genes predicted to be involved in guaiacol catabolism are indicated with an asterisk (*Z*-scores of normalized counts per gene, DESeq2, log_2_FC ≥ 1.5 and adj. *P* < 0.05, **n* *= 4).

To further corroborate our findings and identify any additional early response genes in this genetic pathway that may help regulate guaiacol degradation, we performed a time course transcriptional analysis of Vvg01 bacterial cells growing on mannitol then transferred to grow on guaiacol, with both substrates to be used as sole carbon sources. After switching cells from mannitol to guaiacol M9 liquid medium, we collected cells at 20, 60, and 300 minutes for RNASeq analysis. Our analysis identified 50, 570, and 249 DEGs for time points 20, 60, and 300 minutes post-incubation in guaiacol, respectively (DESeq2, log_2_FC ≥ 1.5 and *P.* adj. < 0.05, S8 File). Like our previous results, we found the same nine genes whose annotation point to enzymes that participate in the complete catabolism of guaiacol to be highly upregulated across all three time points (Fig 3 and Table 2). At every time point, *guaA* and *guaB* were the top two most differentially expressed genes. In terms of genes that could potentially be upstream of the genes responsible for guaiacol degradation, the putative transporters *CC3_GM001949* and *CC3_GM003855* described in the previous section were again observed to be upregulated during time points 60 and 300 minutes. Additionally, we found a couple of putative two-component regulatory systems found to be upregulated upon growth on guaiacol (*CC3_GM002236* and *CC3_GM002237*, and *CC3_GM004155* and *CC3_GM004156*). One system was uniquely upregulated in guaiacol treated cells harvested at 60 minutes and the other at 300 minutes. Further studies are needed to investigate if these genes participate in guaiacol uptake by *G. alkanivorans.*

Furthermore, our time course experiment allowed us to observe expression changes of these genes over time, which reinforce the idea that these genes contribute to utilization of guaiacol as a carbon source. At 20 minutes, although already significantly upregulated, guaiacol degradation genes are at the lowest expression levels among the three time points. At 60 minutes, the expression of these genes is at the highest numbers for this experiment. At 300 minutes, we found that *guaA, guaB,* and the putative genes belonging to the catechol degradation operon are maintained at relatively high expression. However, the genes whose annotation refers to the last four enzymes in the β-ketoadipate pathway, decrease in expression (*CC3_GM1726, CC3_GM1724, CC3_GM1725,* and *CC3_GM1727,* Fig 3 and Table 2). These results indicate guaiacol catabolism gene expression in *Gordonia* cells is dynamic as the cells adapt from an environment where the sugar alcohol mannitol is the sole carbon source to a new environment where guaiacol, an aromatic compound, is the only carbon source.

### *gcoA* participates in the degradation of guaiacol

**Fig 4:**
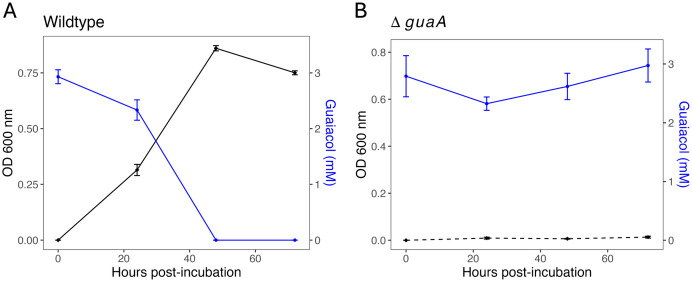
Δ*guaA,* a mutant that lacks a cytochrome P450 monooxygenase, does not grow on guaiacol. *Gordonia alkanivorans* (A) wildtype and (B) Δ*guaA* were grown in M9 medium supplemented with guaiacol (4 mM) as the only carbon source for 72 hours*.* Wildtype (solid black line) showed growth (OD_600 nm_) and guaiacol degradation (blue line) beginning 24 hours post-incubation. Meanwhile, Δ*guaA* (dashed black line) never demonstrated an increase in OD_600 nm_ or significant guaiacol decrease (**n* *= 12)*.* Error bars represent standard error.

To provide further evidence that *guaA* is involved in guaiacol degradation, specifically in the conversion to catechol, we created Δ*guaA*, a *guaA* knockout mutant in *G. alkanivorans* strain Vvg01 (S3 Fig). We tested if Δ*guaA* could grow on M9 + guaiacol (4 mM) liquid medium by breaking down guaiacol alongside its parental wildtype strain. The wildtype strain grew as previously reported with an average OD_600 nm_ of 0.086 at 24 hours post-incubation and an undetectable guaiacol level by our assay (below 0.02 mM) (Fig 4A). However, Δ*guaA* cells never grew and maintained an approximate OD_600 nm_ of 0 throughout the length of the incubation period, 72 hours (Fig 4B). The guaiacol levels in these cultures remained similar to the starting point (0 hours post-incubation) throughout the incubation. These results, along with our RNAseq data, indicate *guaA* is involved in the degradation of guaiacol and its utilization as a carbon source. Because both *guaA* nucleotide sequences in strains Vvg01 and Vvg02 are identical, we expect Vvg02 *guaA* to also play a role in the assimilation of guaiacol.

### Smoke impacts the beta diversity of leaf and berry microbial communities

Because our *Gordonia* strains isolated from the grape leaves demonstrated the ability to use guaiacol as a carbon source to grow, we hypothesized that *Gordonia* spp. may be enriched in grape leaves and berries exposed to smoke, as guaiacol is a major phenolic component in smoke. The phyllosphere is a nutrient-poor environment and any changes in this niche, such as the decrease in moisture and oxygen content and alternate carbon source availability provided by high levels of smoke, can potentially impact the microbiome. To test this, we analyzed the bacterial communities of berries and leaves from full-grown Merlot grapevines (after fruit set) subjected to 36 hours of continuous smoke in the vineyard at the Washington State University Roza Farm. We collected leaf and berry samples before, 36 hours, one week, one month, and two months after smoke treatment. When compared altogether, we found smoke had a significant impact on the alpha diversity of berry and leaf samples (ANOVA followed by Tukey’s Honest Significant Difference test, *adj. P ≤ *0.01). Smoke treated samples resulted in an average Shannon index of 0.12 lower than in the berry control samples (4.71 to 4.59), but about 0.16 higher in leaves (4.65 to 4.81) (S4A and S4B Fig).

To explore the effect of smoke on the grape microbiome community structure, we analyzed the beta diversity via principal coordinates analysis (PCoA) using Bray-Curtis dissimilarity distance.

**Fig 5:**
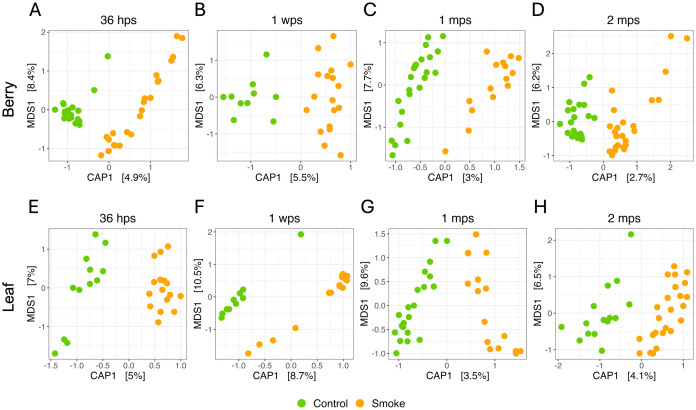
Smoke impacts the beta diversity of grape leaf and berry microbiomes in the vineyard. Beta diversity analysis for **(A-D)** berry and **(E-H)** leaf bacterial communities of smoke-exposed and control (not smoked) *Vitis vinifera* ‘Merlot’ grapevines for 36 hours, one week, one month, and two months post-smoke treatment. Smoke had a significant effect on the berry microbiome at every time point except for 1 mps (Permutational ANOVA, **P* *≤ 0.01). Smoke also had a significant impact on the leaf microbiome across every time point (Permutational ANOVA, **P* *≤ 0.05). Constrained analysis of principal coordinates (CAP) ordination using Bray-Curtis dissimilarity distance (hps = hours post-smoke, wps = weeks post-smoke, mps = months post-smoke).

As with our analysis of alpha diversity, we found that smoke had a significant impact on the berry and the leaf microbiome (S4C and S4D Fig, PERMANOVA, *adj. P ≤ *0.001, *r*^*2*^* *= 0.01256 for berry and *r*^*2*^* *= 0.0131 for leaf). To further explore the effect of smoke on the grape microbiome and to estimate the magnitude of its impact, we analyzed the beta diversity via constrained analysis of principal coordinates (CAP) ordination using Bray-Curtis dissimilarity distance. We found that smoke had a significant impact on the berry microbiome at every time point except for 1 month post-smoke (Permutational ANOVA, **P* *≤ 0.01). Although relatively small, the biggest changes were observed at 36 hours and one week post-smoke treatment with 4.9% and 5.5% of the total variation explained by smoke (Fig 5A-D). Similarly, smoke had a significant effect on the leaf microbiome across every time point (Permutational ANOVA, **P* *≤ 0.05) with time points 36 hours and 1 week post-smoke having the largest effect with 5% and 8.7% of the total variation explained by the smoke treatment (Fig 5E-H), and effect sizes diminishing in magnitude in later weeks.

### Several genera belonging to the Bacilli class are enriched upon smoke exposure

**Fig 6:**
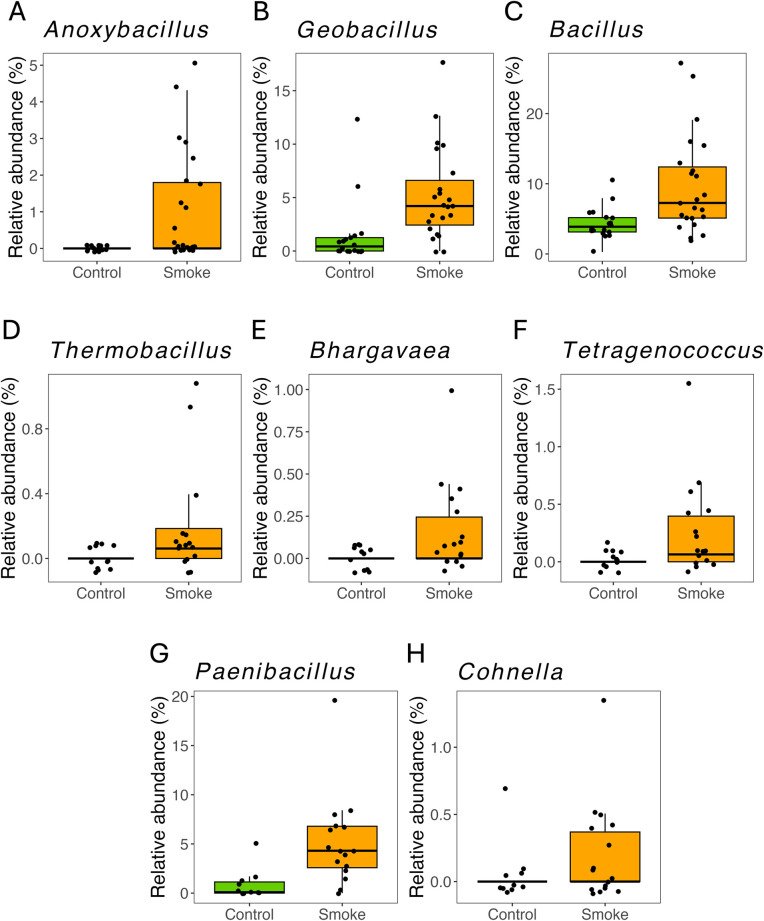
Various genera belonging to the Bacilli class are significantly enriched after exposure to smoke in grape berry and leaf samples. Percent relative abundance for the enriched genera in **(A-C)** berry samples at 36 hours post-smoke, **(D-F)** leaf samples at 36 hours post-smoke, and **(G-H)** 1 week post-smoke (DESeq2, log_2_FC ≥ 1.5 and **P* *< 0.05).

To identify bacterial species that thrived in leaves and berries following smoke treatment, we analyzed those genera that were significantly enriched (DESeq2) within this treatment group compared to the control. Although we detected *Gordonia* ASVs in a small number of berry and leaf samples in this experiment (n = 10 out of 366 leaves and berries), this genus was at very low abundance (average of 0.01% in berry and 0.18% in leaf) within this field site and we did not find a significant increase in its abundance following smoke treatment. Of note, for the eight *Gordonia* ASVs that were detected, none were identified as belonging to the species *alkanivorans*. However, we did detect significant increases in several other Gram positive genera belonging to the Bacilli class within the smoke treatment group at 36 hours and one week after treatment. In berry samples, we found the genera *Anoxybacillus*, *Geobacillus*, and *Bacillus* were enriched with higher relative abundances at 36 hours post-smoke exposure (Fig 6A-C, DESeq2, log_2_FC ≥ 1.5 and **P* *< 0.05). For leaf samples, we observed *Thermobacillus*, *Bhargavaea*, and *Tetragenococcus* to be enriched at 36 hours post-smoke (Fig 6D-F, DESeq2, log_2_FC ≥ 1.5 and **P* *< 0.05). In addition, we found the genera *Paenibacillus* and *Cohnella* to have a significant increase at 1 week post-smoke (Fig 6G and H, DESeq2, log_2_FC ≥ 1.5 and **P* *< 0.05). Overall, these findings show that smoke exposure has a significant impact on specific members of the grape leaf and berry microbiome, and that this impact is characterized by an increase in multiple Bacilli members at early time points.

## Discussion

In this study, we explore the role of wildfire smoke in modulating the grape microbiome and identify two naturally-occurring *Gordonia alkanivorans* strains in the grape phyllosphere that consume guaiacol *in vitro* within 96 hours of incubation. While *Gordonia* spp. are known for their diverse metabolic properties, especially for their ability to degrade xenobiotic compounds, specifically hydrocarbons, and the majority of isolates are from soil [31–33,38–42], there are few reports describing their association with plant hosts [15,43–45]. Previously, a *Gordonia* strain isolated from oil palm tree leaves was shown to degrade isoprene, an air pollutant that reacts with ozone and nitric oxides damaging the atmosphere [46]. In another study, *Gordonia* as a genus was found to be enriched in the rhizosphere of *Sedum alfredii* plants grown in soil with high cadmium levels [44], and evidence from other plant systems suggests that high cadmium can lead to increases in total phenolic compounds within the host tissue [47]. Collectively, these studies demonstrate that, although uncommon, *Gordonia* can be found associated with plant hosts in the phyllosphere and rhizosphere, and that its presence in the plant microbiome may be linked to its specialized metabolic functions. Studying this novel group of bacteria that eliminate potentially detrimental compounds, as well as the enzymes involved, could prove valuable for the development of a variety of biotechnological applications.

Multiple studies have reported the ability of bacteria, including *Gordonia*, to degrade aromatic compounds but few have focused on the specific assimilation of guaiacol and the genes responsible for this process [16–20]. Our genomic data for *G. alkanivorans* and transcriptional analyses during its growth on guaiacol allowed us to identify candidate genes that likely participate in the complete degradation of this compound. Most importantly, it helped us identify *guaA* and *guaB* as the putative *O*-demethylase pair that convert guaiacol to catechol, both which were consistently highly expressed (i.e., top two most upregulated genes) throughout the metabolically active lag and exponential growth phases on guaiacol-containing media. To provide further evidence, we deleted *guaA* and showed these cells could no longer catabolize guaiacol and grow demonstrating GuaA is essential for the consumption and breakdown of guaiacol. *GuaA* and *guaB* and related homologs are not commonly harbored in the genome of prokaryotes but have been identified in a few other Gram positive bacteria. The *G. alkanivorans guaA* and *guaB* identified in our study share genomic organization and similar protein homology with the guaiacol degradation operon and its transcriptional regulator present in *Amycolatopsis* sp. and *Rhodococcus opacus* (*i.e. gcoAB* and *guaR*) [17,18,35]. In *Amycolatopsis*, the *O*-demethylase cytochrome P450 and its reductase pair (GcoAB) were originally described to function in the conversion of guaiacol to catechol, but later studies demonstrated that these enzymes could also demethylate several other aromatic compounds [18]. Here, we tested our two identified *Gordonia* strains for growth on multiple aromatic compounds similar in structure to guaiacol (i.e., phenol, 4-methylguaiacol, eugenol, 4-ethylguaiacol, syringol, and 4-ethylphenol) and demonstrated that neither strain could grow on these compounds. This result suggests that the GuaA cytochrome P450 monooxygenase we identified in *G. alkanivorans* may have high specificity for guaiacol as a substrate. Future *in vitro* experimentation with this family of enzymes could enable a greater understanding of the relative promiscuity of individual cytochrome P450 monooxygenases, and we anticipate that this represents an important knowledge gap for the development of biotechnological tools from these enzymes.

In this study, we also aimed to identify regulatory circuitry upstream of *guaA* and *guaB,* such as receptors and transporters, using longitudinal sampling and RNAseq analysis. From this data, we found only two putative genetic pathways that could play roles as upstream receptors, both of which were two-component regulatory systems found to be upregulated upon growth on guaiacol. To date, two-component systems that recognize aromatic compounds in the extracellular space have yet to be described in the literature. This, in conjunction with the fact that both genes were induced at later time points, suggests that it is unlikely these *Gordonia* strains utilize these putative two-component systems to perceive guaiacol. Additionally, we also found two putative benzoate transporter genes, annotated as BenK and BenE, to be significantly upregulated in both our RNAseq experiments where *Gordonia* cells were harvested during active growth in the lag and exponential phases. The closest annotated relatives of these transporters (by homology) are described as being involved in the uptake of benzoate, an aromatic compound with similar structure to guaiacol [37]. These homologs are from the distantly related Gram negative species *Pseudomonas putida,* which was shown to be capable of growing on benzoate as the sole carbon source [36,37]. We speculate that the two transporter genes identified in our own study likely participate in the intracellular uptake of guaiacol by *G. alkanivorans* Vvg01 and Vvg02, though future genetic and *in vitro* experimentation will be needed to confirm this hypothesis.

Another important goal of this study was to determine if wildfire smoke exposure impacts the grape microbiome. The 16S rRNA microbial community analysis of Merlot grapevines grown in the presence of smoke in a vineyard setting determined that smoke does have a significant effect on community composition of leaves and berries. Following our discovery of *G. alkanivorans* Vvg01 and Vvg02 and their ability to degrade guaiacol, we hypothesized that the aromatic compounds found in smoke and the nutrient poor environment in the phyllosphere may lead to an enrichment of *Gordonia* and related genera during smoke events typical of wildfires. However, the data from our simulated smoke event in the field did not suggest a significant enrichment in this genus. This may be due to the fact that samples from our experiment only contained trace levels of *Gordonia*, possibly insufficient to allow us to observe whether smoke treatment leads to enrichment of this genus. Alternatively, the specific *Gordonia* species present in this field experiment may not possess the capability to degrade these compounds; 16S rRNA often does not afford species level resolution, and in this instance the closest relatives of the identified *Gordonia* likely belonged to species other than *alkanivorans*.

Interestingly, we did identify a strong and significant enrichment of multiple genera belonging to the Bacilli class. Bacterial species within the Bacilli class are known to be fast growers, possibly enabling them to respond rapidly to conditions that favor their growth. As Bacilli are also capable of surviving in extreme environments, including those with high heat and low moisture [48], it is plausible that native Bacilli residents in the grape microbiome develop a competitive advantage during smoke exposure. Alternatively, these Bacilli may be brought into the grape microbiome with the smoke itself; recent results from the human health field have found that Bacilli are capable of being aerosolized within cigarette smoke [49]. Interestingly, *Bacillus* and related *Paenibacillus* are also among the most prevalent in the lung microbiomes of smokers, suggesting another connection between this lineage and smoke exposure [49,50]. Future experimentation with Bacilli and related lineages may help resolve the causes of this observed phenomenon, and explore the extent to which these species are also capable of consuming volatile phenols found in smoke.

In conclusion, smoke taint is a new and significant problem in the US costing wine grape growers and winemakers millions of dollars in recent years. This industry is essential for the viability of the agricultural economies of the states Washington, Oregon, and California and growers and winemakers are in dire need of solutions to this problem. Bacteria have a breadth of metabolic capabilities making them good candidates for the exploration of novel enzymatic applications. Because of this, we took a microbial approach in this study as a potential future solution to help eliminate unwanted volatile phenols such as guaiacol in wine berries and must. It is clear that many other compounds besides guaiacol contribute to smoke taint (*i.e.,* thiophenols and glycosides of multiple phenols) and that additional studies aimed at identifying enzymes that can degrade these metabolites are warranted; the current study provides a roadmap for these future efforts The identification of novel, grape-associated bacteria with the ability to consume guaiacol, and the enzymes responsible for this degradation, opens the door to translating microbial capabilities to industrial solutions for this critical challenge.

## Supporting information

S1 Fig Two grape leaf epiphytes grow in liquid M9 medium supplemented with guaiacol (4 mM) as the only carbon source. (A) Two bacterial isolates were selected from a complex leaf epiphyte community for their ability to grow on guaiacol and their colony morphology shown on trypticase soy agar medium. (B) Bacterial cultures in M9 medium with guaiacol (only carbon source, 4 mM) show turbidity after four days incubation at 30°C, 250 rpm. (C) Bacterial growth (OD_600 nm_) quantification over time at 30°C, 250 rpm (*n *= 12). Error bars represent standard error.
(TIF)

S2 Fig *Gordonia alkanivorans* Vvg01 and Vvg02 do not grow in liquid M9 medium supplemented with either phenol, 4-methylguaiacol, eugenol, 4-ethylguaiacol, syringol, or 4-ethylphenol (0.5 g/L) as sole carbon sources after seven days incubation at 30°C, 250 rpm (*n *= 3). (TIF)

S3 Fig Confirmation of *guaA* knockout and replacement with gentamycin resistance cassette. Lane ‘L’, GeneRuler 1 kb Plus DNA ladder (Thermo Scientific, Cat. No. 1333); lanes 1–24 colonies screened for knockout; lane ‘(+)’, pCC1 as positive control; lane ‘(-)’, wildtype gDNA as negative control. Lane 17 and positive control showed a single band of size ~1.7 kb corresponding to a colony with the correct *guaA* knockout. Negative colonies and negative control showed at least one band of size ~2.2 kb. PCR products were generated with primers ‘omCYP450_1_fwd’ and ‘omCYP450_6_rev.’ 1.0% agarose gel was used to separate PCR products.
(TIF)

S4 Fig Alpha diversity measure using Shannon index for (A) berry and (B) leaf bacterial communities of smoke-exposed and control (not smoked) *Vitis vinifera* ‘Merlot’ grapevines for all time points: before smoke, 36 hours, one week, one month, and two months post-smoke treatment. Principal coordinates analysis of Bray-Curtis distance as a measure of beta diversity for (C) berry and (D) leaf bacterial communities of smoke-exposed and control grapevines for all time points (hps = hours post-smoke, wps = weeks post-smoke, mps = months post-smoke).
(TIF)

S1 File Read mapping results for Vvg01 (OM) and Vvg02 (OD) in RNAseq experiment no. 1. (XLSX)

S2 File Read mapping results for Vvg01 in RNAseq experiment no. 2. (XLSX)

S3 File Strains, plasmids, and primer sequences used in this study. (XLSX)

S4 File Synthetic DNA fragments used in this study. (XLSX)

S5 File Differentially expressed genes for Vvg01 in RNAseq experiment 1. (XLSX)

S6 File Differentially expressed genes for Vvg02 in RNAseq experiment 1. (XLSX)

S7 File GuaAB protein sequence percent similarity. *G. alkanivorans* Vvg01 and Vvg02 GuaAB protein sequence similarity to previously characterized *R. opacus* PD630 and *Amycolatopsis* sp. ATCC39116 GcoAB. *R. opacus* PD630 GcoAB accession numbers are WP_005251375.1 and WP_025433214.1. *Amycolatopsis* sp. ATCC39116 GcoAB accession numbers are WP_020419855.1 and WP_020419854.1.
(XLSX)

S8 File Differentially expressed genes for Vvg01 in RNAseq experiment 2. (XLSX)

S9 File Materials and methods continued. (DOCX)

S1 Raw S1 raw images. (PDF)
