# Supplementary figures and images for: Bacteria isolated from the grape phyllosphere capable of degrading guaiacol, a main volatile phenol associated with smoke taint in wine
Source: PLoS One. 2025 Oct 1;20(10):e0331854. doi: 10.1371/journal.pone.0331854 (PMC12488008; doi:10.1371/journal.pone.0331854)

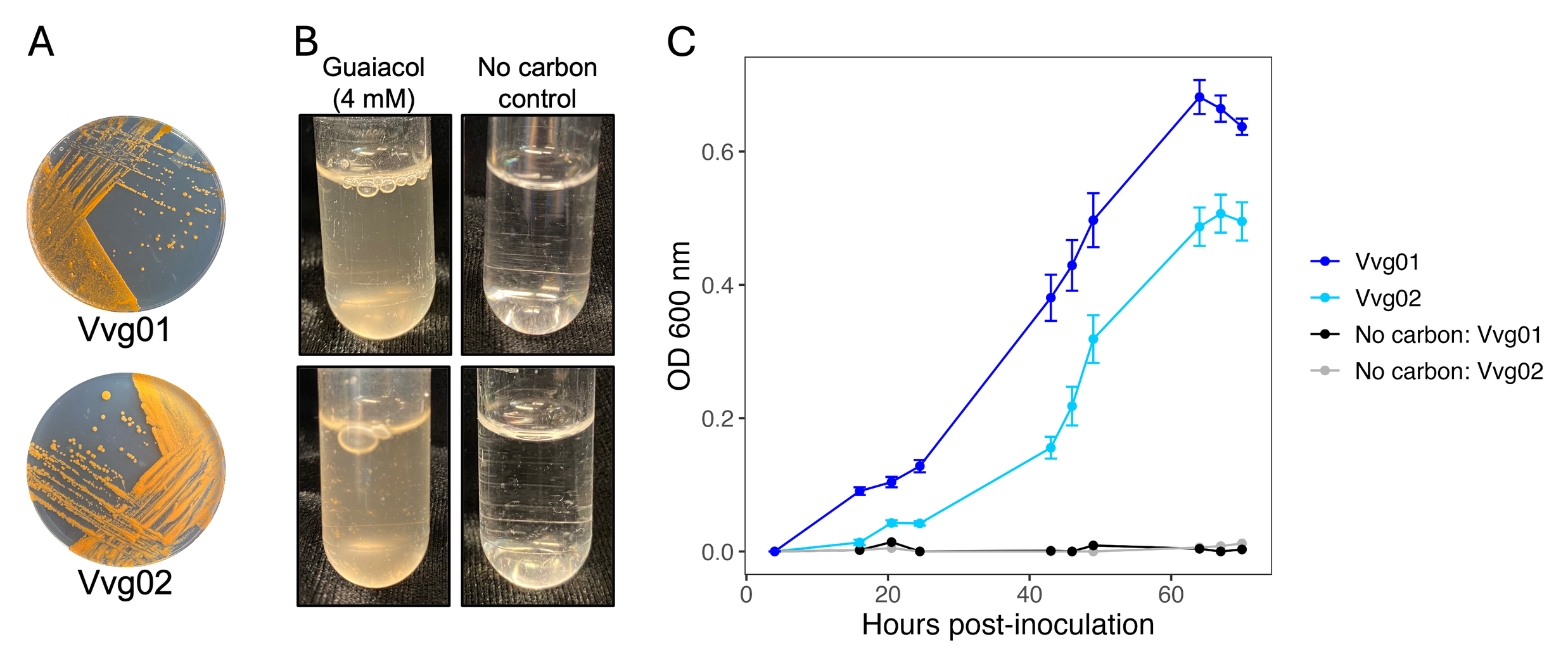

Supplement: S1 Fig — (A) Two bacterial isolates were selected from a complex leaf epiphyte community for their ability to grow on guaiacol and their colony morphology shown on trypticase soy agar medium. (B) Bacterial cultures in M9 medium with guaiacol (only carbon source, 4 mM) show turbidity after four days incubation at 30°C, 250 rpm. (C) Bacterial growth (OD600 nm) quantification over time at 30°C, 250 rpm (n = 12). Error bars represent standard error. (TIF) [file pone.0331854.s001.tif]

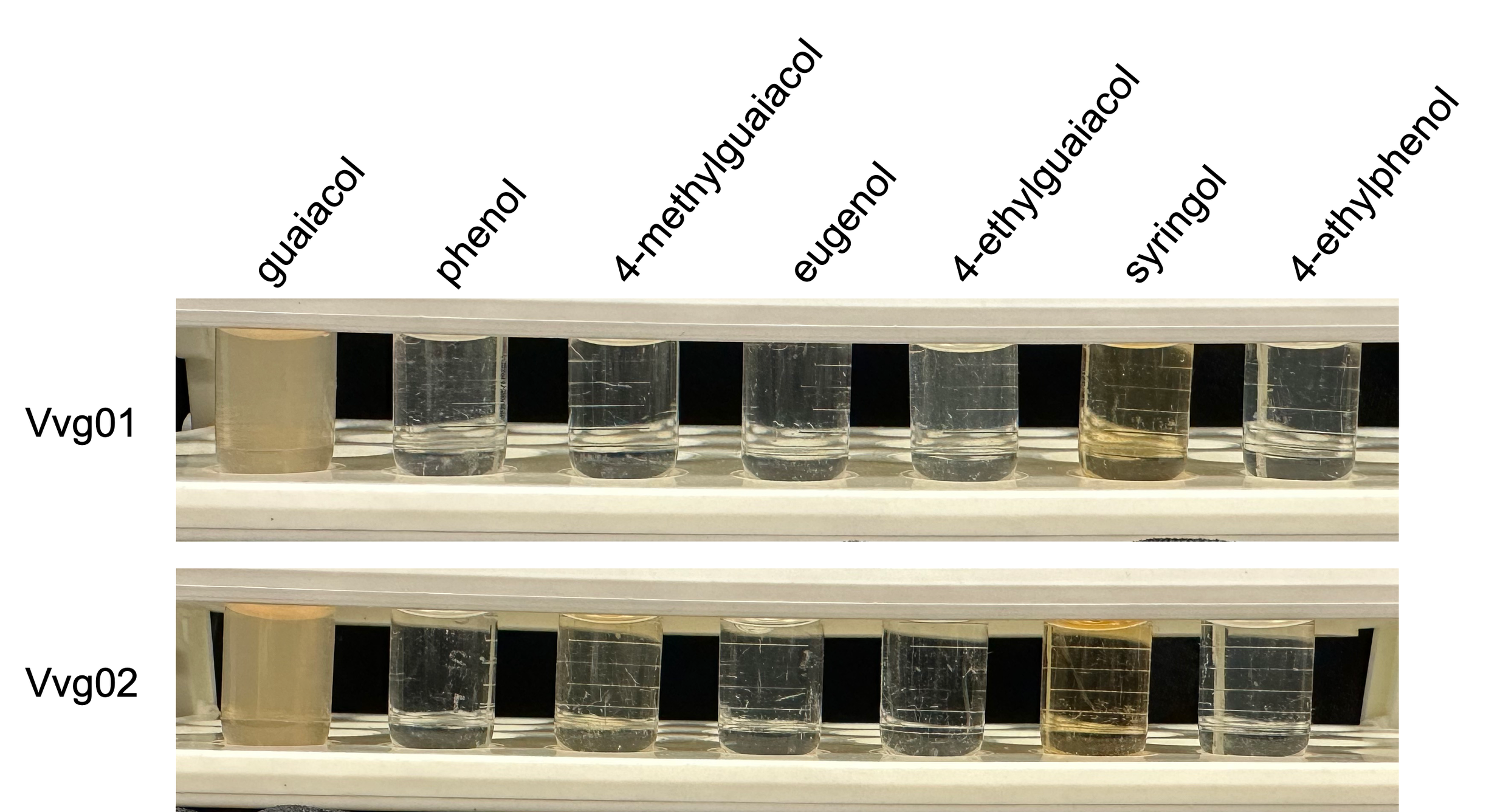

Supplement: S2 Fig — (TIF) [file pone.0331854.s002.tif]

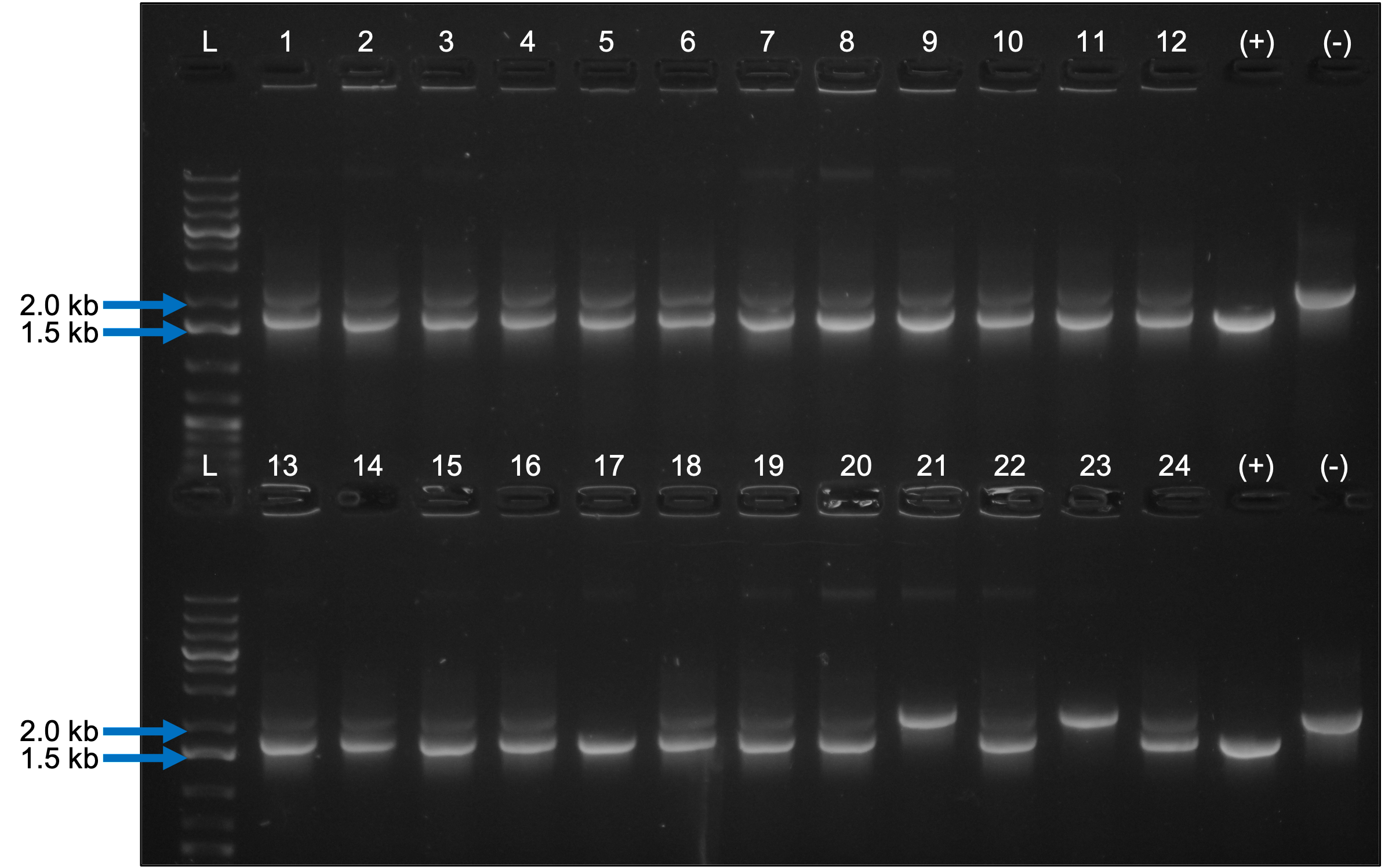

Supplement: S3 Fig — Lane ‘L’, GeneRuler 1 kb Plus DNA ladder (Thermo Scientific, Cat. No. 1333); lanes 1–24 colonies screened for knockout; lane ‘(+)’, pCC1 as positive control; lane ‘(-)’, wildtype gDNA as negative control. Lane 17 and positive control showed a single band of size ~1.7 kb corresponding to a colony with the correct guaA knockout. Negative colonies and negative control showed at least one band of size ~2.2 kb. PCR products were generated with primers ‘omCYP450_1_fwd’ and ‘omCYP450_6_rev.’ 1.0% agarose gel was used to separate PCR products. (TIF) [file pone.0331854.s003.tif]

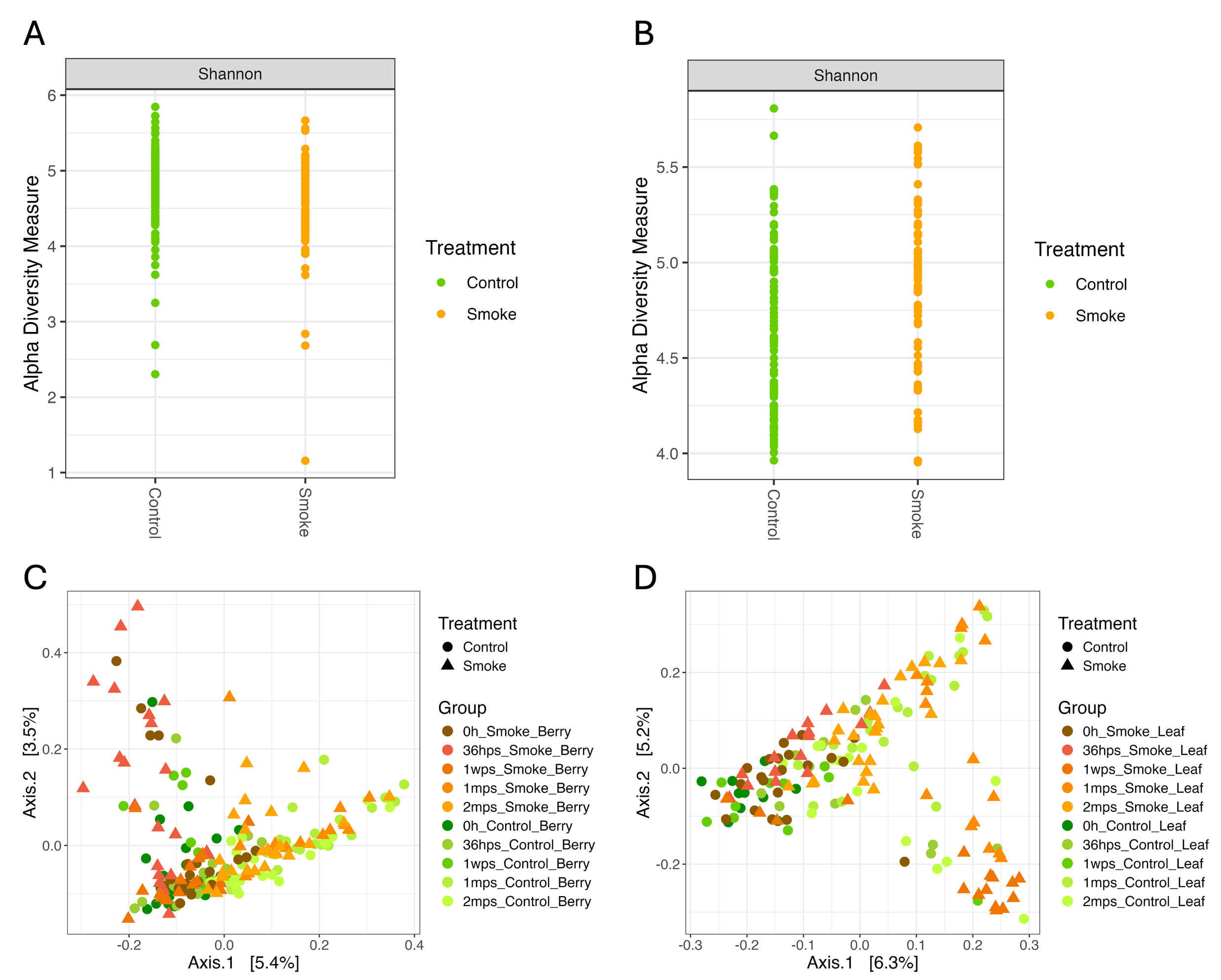

Supplement: S4 Fig — Principal coordinates analysis of Bray-Curtis distance as a measure of beta diversity for (C) berry and (D) leaf bacterial communities of smoke-exposed and control grapevines for all time points (hps = hours post-smoke, wps = weeks post-smoke, mps = months post-smoke). (TIF) [file pone.0331854.s004.tif]

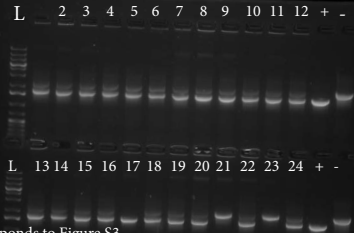

Corresponds to Figure S3

Supplement: S1 Raw — (PDF) [file pone.0331854.s014.pdf]
