# Supplementary material for: Bacteria isolated from the grape phyllosphere capable of degrading guaiacol, a main volatile phenol associated with smoke taint in wine
Source: PLoS One. 2025 Oct 1;20(10):e0331854. doi: 10.1371/journal.pone.0331854 (PMC12488008; doi:10.1371/journal.pone.0331854)
Supplement: S9 File — (DOCX) [file pone.0331854.s013.docx]

**S9 File. Materials and methods continued.**

***guaA* gene knockout *continued***

Overlap extension PCR was used to join 5’ and 3’ flanking regions adjacent to the target gene *guaA* in Vvg01 with a gentamicin resistance gene to create a deletion construct. Flanking regions were 500 bp in length to facilitate homologous recombination within the Vvg01 chromosome to remove *guaA*. Resulting overlap PCR amplicon was cloned into pCR8/GW/TOPO vection and transformed into *E. coli* TOP10 cells (Invitrogen, Cat. No. K4500J10). Deletion construct was amplified from plasmid using primers ‘cyp450-OM_hifi_fwd’ and ‘cyp450-OM_hifi_rev’ and then inserted into the pJE382 vector using NEBuilder HiFi DNA Assembly Master Mix (New England Biolabs, Cat. No. E5520S) to create ‘pCC1.’ This resulting plasmid was transformed into *E. coli* NEB 5-alpha cells and verified colony PCR and Sanger sequencing.

Construction of plasmids pMTV1821 and pMTV1823 was performed and confirmed by GenScript (Piscataway, NJ, USA). Identified methyltransferases in Vvg01 were cloned into pMTV210 to be expressed from an arabinose-inducible promoter once inserted in *E. coli*. Gene sequences expressing methyltransferases HaeIIIM_1-CC3 [PGBNALGA_00822] and HaeIIIM_2-CC3 [PGBNALGA_02904] were codon optimized to *E. coli* K12 and synthesize by GenScript (Nanjing, China). Synthesized DNA fragments are listed in S4 File.

Genes expressing *Gordonia* *alkanivorans* Vvg01 methyltransferases were integrated into *E. coli* via the Serine-integrase Assisted Genome Engineering (SAGE) method (50,51). To generate *E. coli* strain AG13554, plasmid pMTV1821 that expresses HaeIIIM_2-CC3 [PGBNALGA_02904] was integrated in *E. coli* strain AG5589. Briefly, a temperature sensitive plasmid expressing the BXB1 integrase (pLAR074) was used, and 200 ng of each plasmid was transformed into 50 μl of AG5589 electrocompetent cells. The transformation was recovered in 1 mL SOC at 30°C for 40 minutes to allow replication of pLAR074 and transient expression of BXB1 recombinase and then moved to 42°C for another 40 minutes for plasmid curing. The transformation was plated onto LB (Miller) supplemented with 30 μg/mL kanamycin and incubated at 37°C for 24 hrs. Colonies were picked and grown on 2 mL LB with 30 μg/mL kanamycin and incubated at 42°C overnight. Colony PCR with primers ‘oMTV2671/oMTV2672’ and ‘oMTV24/oMTV27’ were used to confirm integration of HaeIIIM_2-CC3 [PGBNALGA_02904] and the kanamycin marker in AG5589. For the removal of the kanamycin marker, competent cells were made from the strain confirmed above. Another temperature sensitive plasmid expressing the ɸC31 integrase (pLAR047) was transformed in 50 μl of competent cells and recovered in SOC at 30°C. The transformation was plated on LB with 100 μg/mL carbenicillin. Colonies were picked into LB broth and grown at 42°C overnight to cure pLAR047. Then, 1 μl of the outgrowth was streaked on LB plates and grown overnight at 37°C. Colonies were then patched onto LB with kanamycin 30 μg/mL, LB with 100 μg/mL carbenicillin and plain LB to screen for the loss of the kanamycin marker and loss of the helper plasmid pLAR047. Colony PCR was used to confirm HaeIIIM_2-CC3 [PGBNALGA_02904] and the loss of the kanamycin marker, the resulting strain is AG13554. Using the procedure described above, electrocompetent cells were made from AG13554 to integrate pMTV1823 HaeIIIM_1-CC3 [PGBNALGA_00822]. For this strain, pLAR051 was used as the helper recombinase, resulting in strain AG13708, which has both methyltransferases integrated into the *E. coli* chromosome under the control of arabinose-inducible promoters.

Methylation of pCC1 was induced by growing *E. coli* AG13708 cells in LB supplemented with arabinose (1 mM) (52). 1 μg of pCC1 (methylated in *E. coli* AG13708) was transformed into 100 μL *G. alkanivorans* Vvg01 cells via electroporation with a 0.2 cm cuvette (BioRad, Cat. No. 164-2086) and a MicroPulser electroporator (BioRad, Cat. No. 1652100) with settings 3.0 kV, 600 Ω, and 25 μF. 1 mL LB was immediately added to the cells following a recovery incubation overnight at 30°C, 250 rpm. Cells were spread-plated on LB agar plates containing kanamycin (50 μg/mL). Colonies appeared after 3 to 4 days of incubation.

The screening protocol used was adapted from Hmelo et al. (53). pJE382 suicide vector was used. This vector contains pUC origin which doesn’t allow replication in Actinomycetes, a kanamycin selection marker, and *sacB* for sucrose counter selection. The colonies that appeared on LB kanamycin plates were subcultured again on LB kanamycin plates for single colony isolation. These plates were incubated for 7 days at 30°C. A minimum of two colonies were picked from each plate and subcultured for single colony isolation on TSA plates with 10% sucrose w/v and incubated for five days. Those colonies that produced a “patchy”, low growth, first streak on the TSA sucrose plates were selected and two to three colonies from each were subcultured once again on TSA sucrose plates. The single colonies generated at this point were used for colony PCR and Sanger sequencing to confirm knockout.

**Smoke exposure and sample preparation *continued***

The protocol developed by Inglis et al. was followed to extract DNA from berry and leaf tissues with some modification (54). Ground samples were washed twice with 1 mL sorbitol pre-wash buffer, centrifuged at 10,000xg for 5 min., and the supernatant was discarded. 800 μL of warm CTAB buffer and 3 μL RNase A (20 mg/mL, Cat. No. T3018L, New England Biolabs) were added to the tissue pellet and vortexed to resuspend the pellet. The sample was incubated in a 65 °C water bath for 30 min. and vortexed every 5-10 min for 20 s. Samples allowed to cool at room temperature for 5 min. 500 μL chloroform:isoamyl alcohol (24:1, Cat. No. C0549, Millipore-Sigma) were added to the sample, vortexed vigorously for 1 min. and centrifuged at 18,000xg for 5 min. The aqueous phase was transferred to a new 1.5 mL microcentrifuge tube. 1/10 volume 3 M sodium acetate (Cat. No. S2889, Millipore-Sigma) pH 5.2 was added to the sample and mixed. 2/3 volume isopropanol (Cat. No. I9516, Millipore-Sigma) were added and samples were incubated at -80 °C for 30 min. to precipitate DNA. Samples were centrifuged at 18,000xg for 10 min. and their supernatant was discarded. The DNA pellet was incubated with 1 mL 70% ethanol for 30 min. at room temperature. The sample was centrifuged at 18,000xg for 2 min. and its supernatant was discarded. The DNA pellet was dried for 10-15 min. and resuspended with 60 μL sterile, nuclease-free water. DNA concentration was measured with a Qubit 3.0 fluorometer (Invitrogen, Waltham, MA, USA) and Qubit 1X dsDNA Broad Range Assay Kit (Cat. No. Q33266, Invitrogen).
